# Gastropathy associated with lanthanum phosphate deposition that was endoscopically tracked for 3 years. A case report

**DOI:** 10.1186/s12876-020-01424-7

**Published:** 2020-08-31

**Authors:** Akiko Ohno, Jun Miyoshi, Hidesato Tanabe, Mitsunori Kusuhara, Masao Toki, Tomohiro Chiba, Hiroaki Shimoyamada, Junji Shibahara, Tadakazu Hisamatsu

**Affiliations:** 1 Department of Gastroenterology and Hepatology, Kyorin University School of Medicine, 6-20-2 Shinkawa, Mitaka-shi, Tokyo, 181-8611 Japan; 2 Department of Gastroenterology, Kosei Hospital, 2-25-1 Wada, Suginami-ku, Tokyo, Japan; 3 Department of Pathology, The Cancer Institute Of JFCR, 3-8-31, Ariake, Koto, Tokyo, 135-8550 Japan; 4 Department of Pathology, Kyorin University School of Medicine, 6-20-2 Shinkawa, Mitaka-shi, Tokyo, 181-8611 Japan

**Keywords:** Lanthanum carbonate, Dialysis, Mucosal deposition, Stomach, Histiocyte

## Abstract

**Background:**

With the recent increased use of lanthanum carbonate, several cases of lanthanum phosphate deposition to gastric mucosa in dialysis patients have been reported. However, the endoscopic appearance of the early-stage lesion and the over-time alterations of endoscopic findings due to the progression of lanthanum phosphate deposition remain unclear.

**Case presentation:**

An 80-year-old man receiving dialysis and taking lanthanum carbonate as a phosphate binder over a 4-year period underwent upper gastrointestinal endoscopy four times beginning 1 year after initiation of treatment. The first endoscopic examination (after 1 year of exposure to lanthanum carbonate) revealed rough mucosa with a few areas of white granular mucosa. Over the 3 years of endoscopic follow-up, the white granular mucosa spread and multiple erosions appeared. Histopathological findings of biopsy specimens from an erosion showed extensive infiltration by histiocytes containing deposits. Scanning electron microscopy-energy dispersive X-ray spectroscopy (SEM-EDX) revealed that the presence of the deposits containing phosphorus and lanthanum in the gastric mucosa.

On the basis of these results, the patient was diagnosed with gastropathy associated with lanthanum phosphate deposition.

**Conclusions:**

Over a 3-year period, endoscopic findings associated with lanthanum deposition gradually changed and expanded from the early stage.

## Background

Lanthanum carbonate is commonly used in dialysis patients to improve the hyperphosphatemia associated with chronic kidney disease. Specific gastric mucosal findings associated with the deposition of lanthanum have been reported in recent years in patients taking lanthanum carbonate [1–4]. Various endoscopic findings, such as white granular mucosa, ulcers, and erosion, are associated with lanthanum deposition; however, there have been no reports of changes over time in endoscopic findings in a single case from the onset of these changes.

We herein report the gradual changes in endoscopic findings resulting from lanthanum deposition over a 3-year period in a patient receiving ongoing oral administration of lanthanum carbonate.

## Case presentation

An 80-year-old Japanese man diagnosed with chronic renal failure secondary to type 2 diabetes received dialysis for 4 years and took lanthanum carbonate at dose 750 mg/day orally as a phosphate binder during this 4-year period. The patient underwent upper gastrointestinal endoscopy 1 year after initiation of lanthanum carbonate because of epigastric discomfort.

**Fig. 1:**
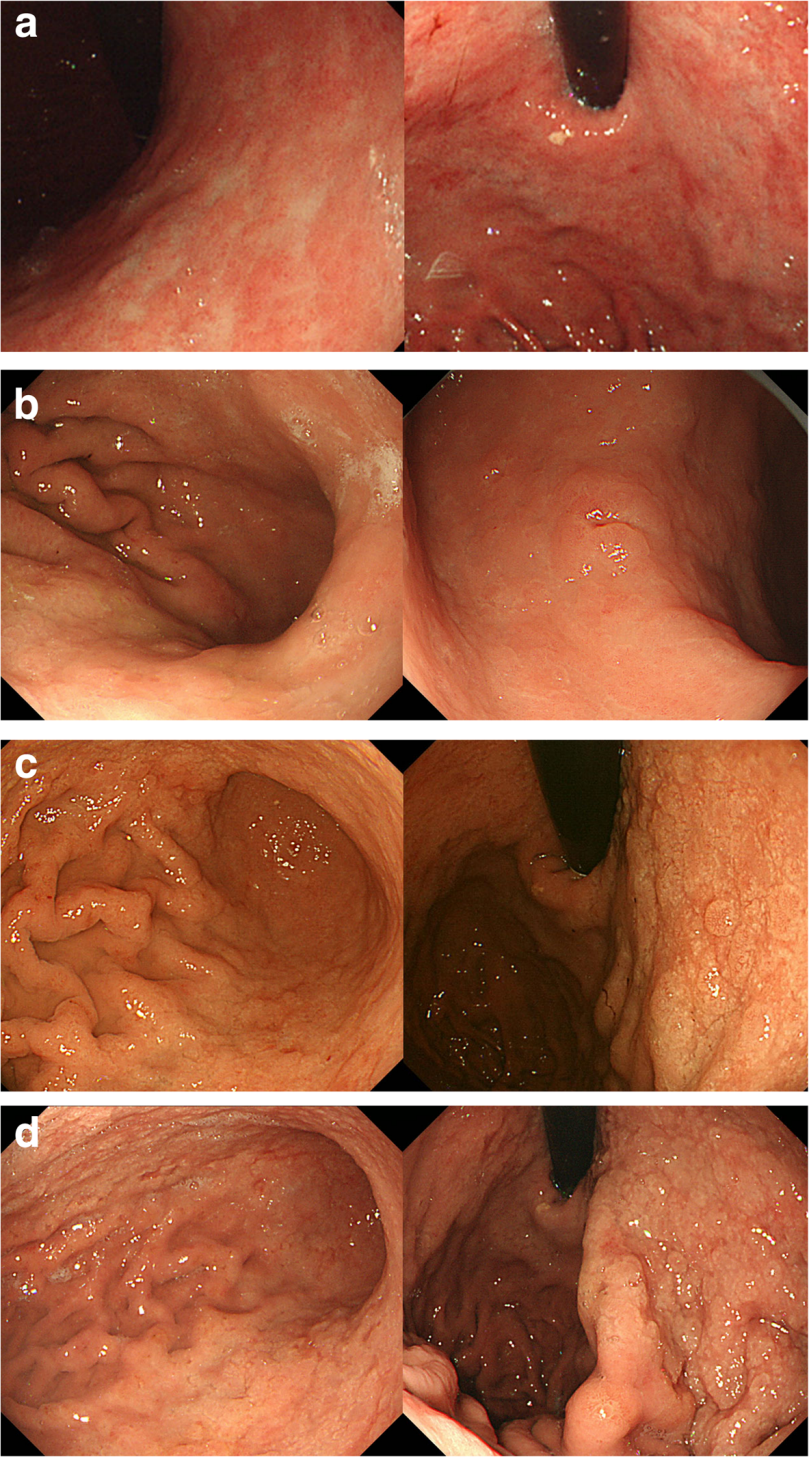
Changes in endoscopic findings over 3 years. **a** One-year exposure to lanthanum: The mucosa was rough compatible with atrophic gastritis but a little white granular mucosa was observed in the lesser curvature of the gastric body. **b** Two-year exposure to lanthanum: The white granular mucosa was clearly observed in lesser curvature and the multiple erosions suggesting the presence of inflammation appeared in the greater curvature of the lower gastric body. **c** Three-year exposure to lanthanum: The white granular mucosa spread widely and erosions also remained. **d** Four-year exposure to lanthanum: Endoscopic alterations observed at the third examination were further widespread, and more conspicuous

The endoscopic findings at this initial endoscopy included rough mucosa with a few white granular lesions in the lesser curvature of the gastric body with the atrophic change (Fig. 1a). After 2 years of exposure to lanthanum, the white granular mucosa was clearly observed in the lesser curvature and had slightly expanded to the greater curvature of the lower gastric body and antrum. Multiple erosions were also observed in the greater curvature of the lower gastric body suggesting the exacerbation of inflammation (Fig. 1b). After 3 years of exposure to lanthanum carbonate, erosions remained and the white granular mucosa had thickened and expanded to the upper gastric body (Fig. 1c). After 4 years of exposure, the endoscopic findings described above became more widespread and conspicuous. The gastric mucosa with chronic inflammation was endoscopically observed thicker than at previous examinations (Fig. 1d).

**Fig. 2:**
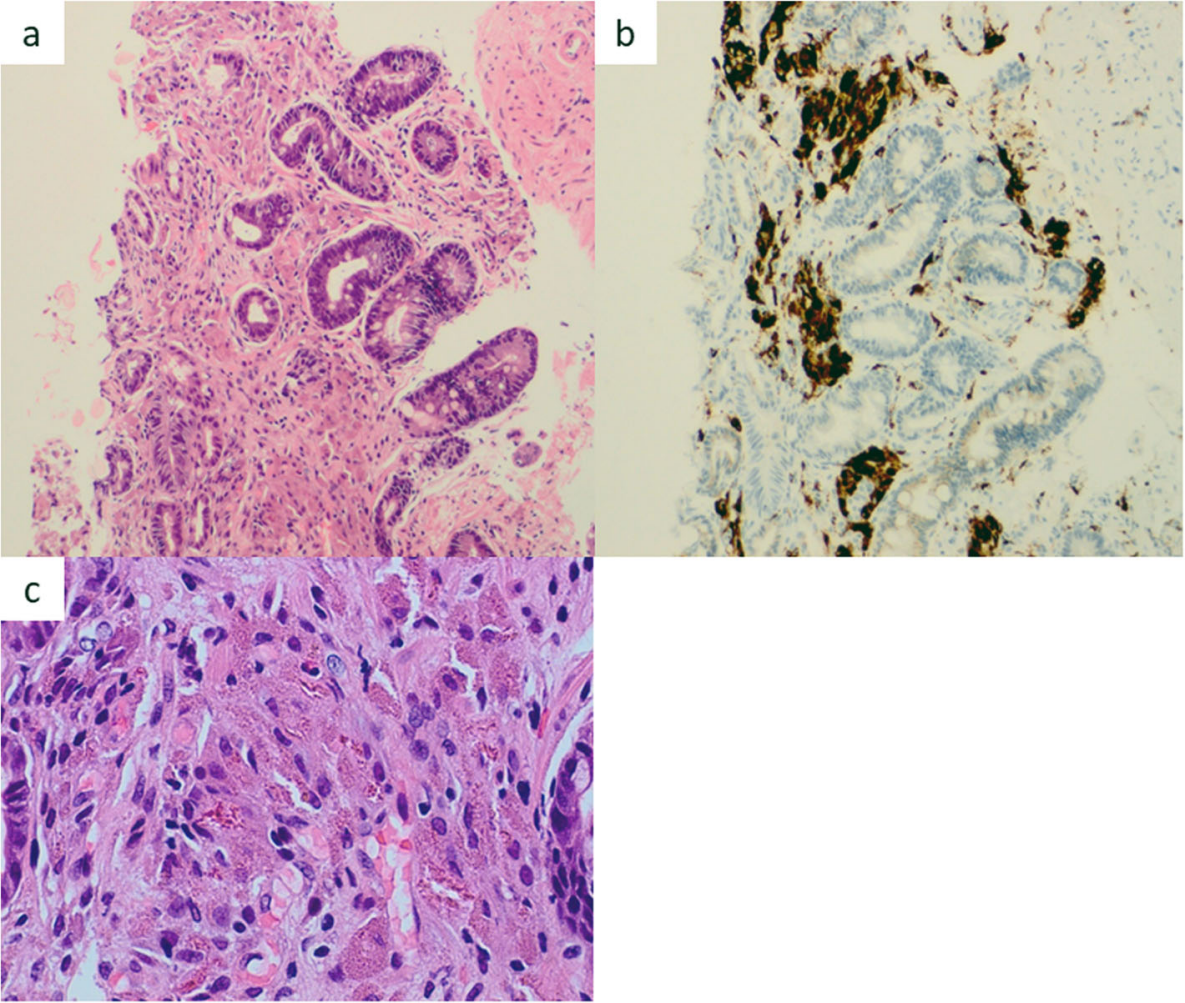
Histologic features of biopsy specimens from erosion. In the foveolar epithelium, atrophic change and replacement with intestinal metaplasia were observed. Many histiocytes with granular or crystalline eosinophilic deposits inside infiltrated extensively, consistent with the presence of inflammation. (HE staining) (**a**, **c**). These histiocytes were immunoreactive to CD68 (**b**)

Histopathological findings on biopsy of erosion in greater curvature showed atrophic mucosa, intestinal metaplasia, and regenerative change. Many histiocytes containing granular or crystalline eosinophilic deposits were found extensively infiltrating the lamina propria, consistent with the presence of inflammation (Fig. 2).

**Fig. 3:**
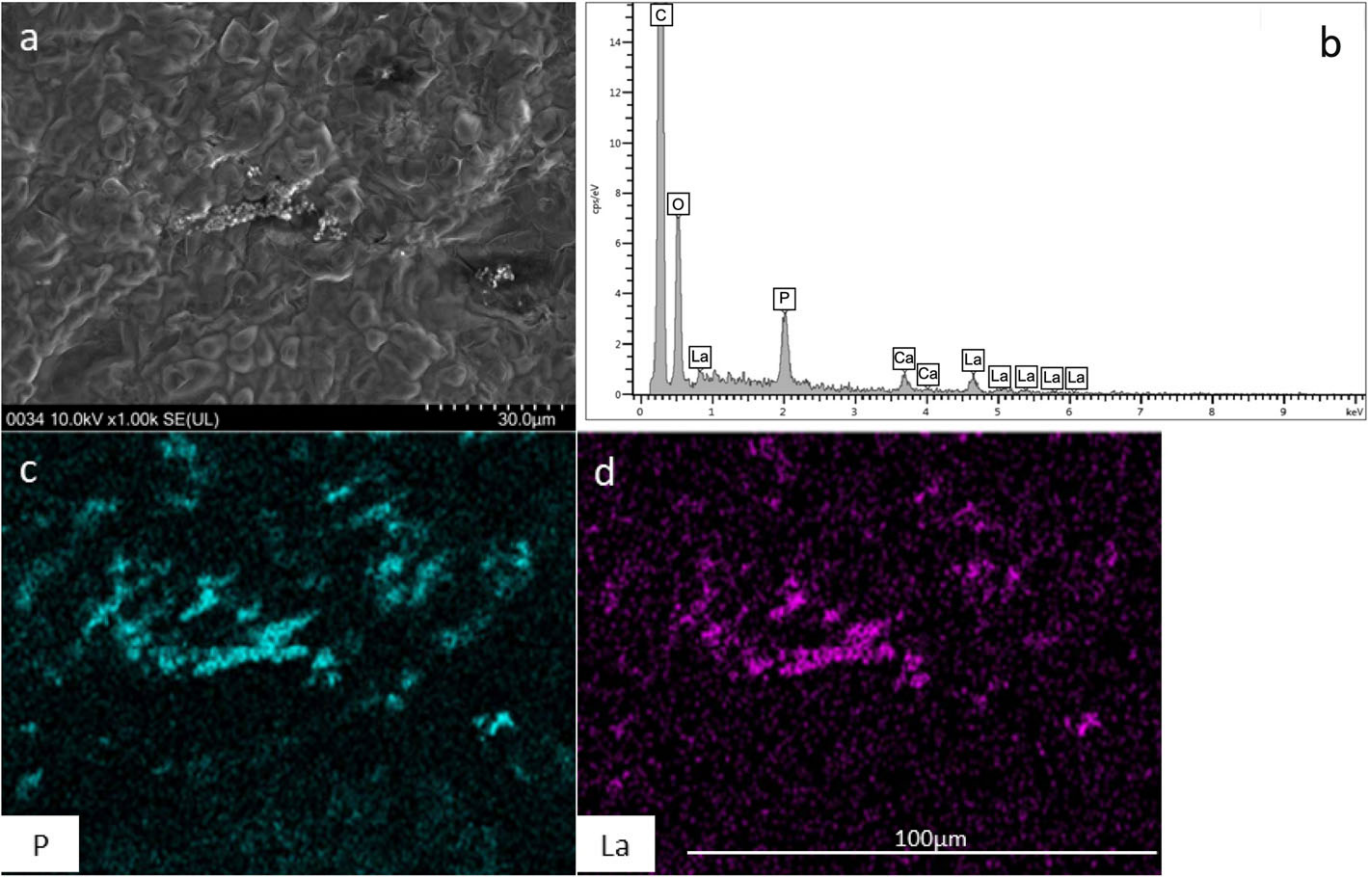
Analysis with scanning electron microscopy–energy dispersive X-ray spectroscopy (SEM-EDX). **a** Deposits in histiocytes were observed as bright materials. **b** Phosphorus and lanthanum ware detected in deposits. **c** Element mapping on the SEM images of phosphorus. **d** Element mapping on the SEM images of lanthanum

Scanning electron microscopy-energy dispersive X-ray spectroscopy (SEM-EDX) identified the deposition of phosphorus and lanthanum in gastric mucosa. Figure 3a shows a representative picture of the scanning electron microscopy of deposits in histiocytes as bright materials. The spectrums corresponding to phosphorus and lanthanum were detected (Fig. 3b). The presence of phosphorus and lanthanum in the high-luminance substance depositions were observed (Fig. 3c and d).

**Fig. 4:**
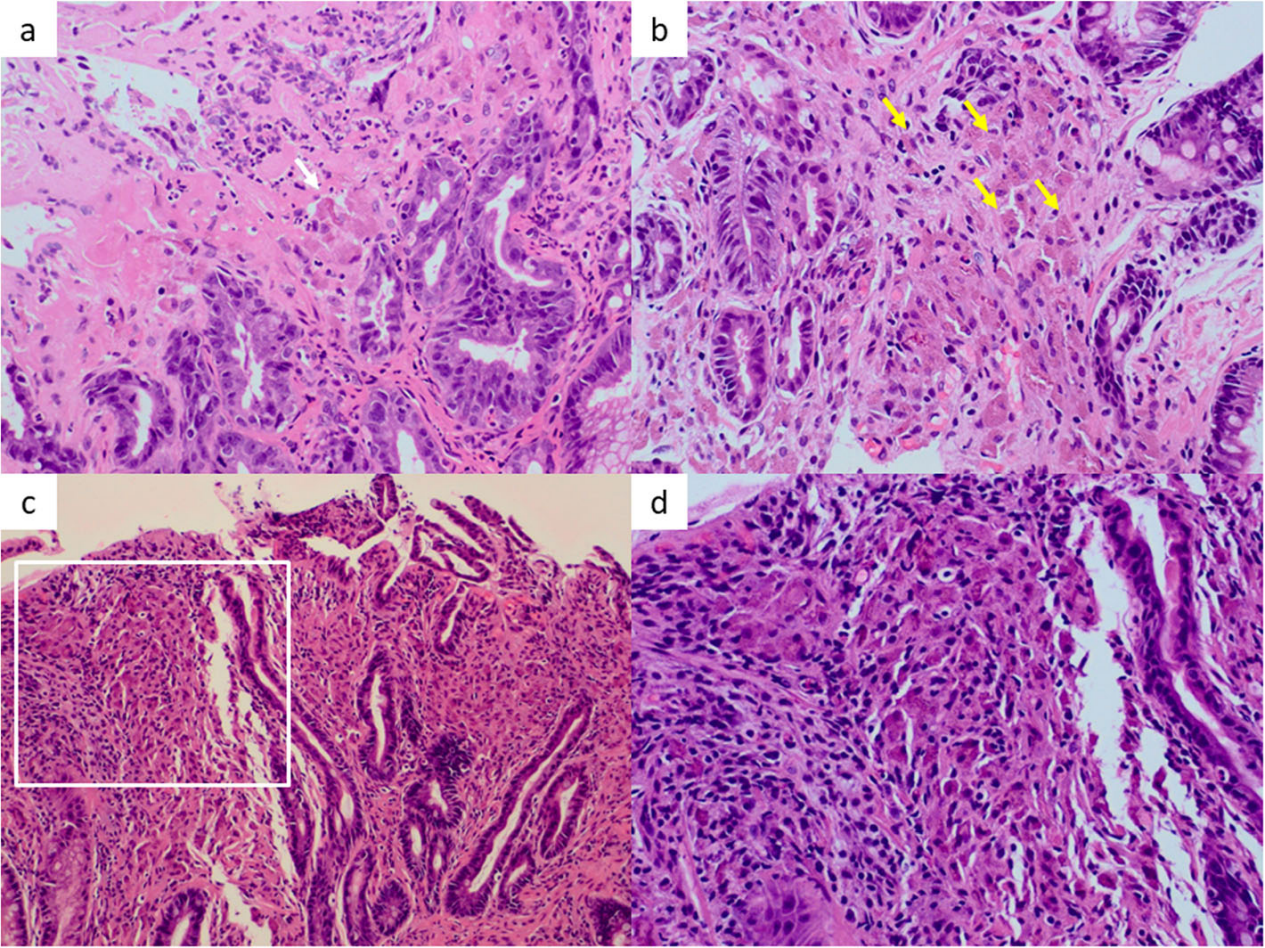
Histological change of biopsy specimens. **a** Two-year exposure to lanthanum: Only a few histiocytes containing crystalline eosinophilic deposits were observed (white arrow). **b** Three-year exposure to lanthanum: Granular or crystalline eosinophilic deposits and infiltration of histiocytes corresponding to the inflammation increased in lamina propria (yellow arrow). **c**, **d** Four-year exposure to lanthanum: The crypt density reduced, and chronic gastritis, regenerative changes, and intestinal metaplasia were more apparent. The deposits and histiocytes that phagocytose them more increased. Panel (**d**) shows the high-magnification image of the area of (white square in (**c**))

We took biopsy specimens from erosion in greater curvature over time (Fig. 4). It was not possible to diagnose that the deposits were lanthanum at the first biopsy, but, by retrospectively reviewing, deposits were observed from the first biopsy specimen. Although biopsies were not taken from the exact identical lesion, the infiltration of histiocytes gradually increased in the lamina propria of gastric mucosa, and granular deposits obviously increased. The crypt density was reduced, and chronic gastritis, regenerative changes, and intestinal metaplasia were more apparent over time.

Because the patient has no abdominal symptoms, gastrointestinal endoscopy has been continued annually for follow-up observation. The patient signed the explanation and consent forms in the informed consent process for endoscopic examination.

## Discussion and conclusions

Lanthanum carbonate is a therapeutic agent for hyperphosphatemia and is widely used for the management of chronic renal failure in dialysis patients. Lanthanum carbonate has been available in the UK since 2007 and in Japan since 2009. Lanthanum carbonate strongly binds with phosphate ions in the intestinal tract to form minimally soluble lanthanum phosphate. Although the absorption rate of lanthanum from the gastrointestinal tract to the bloodstream is considered safe at less than 0.00127% [5], there have been several reports of lanthanum deposition in the gastrointestinal mucosa since 2015. Endoscopic findings include white thickening along gastric mucosal folds, fold-like white thickening, annular white thickening, a number of minutes and irregular white spots, erosion, polyps, and ulcers. Among the various findings, white lesions are regarded as relatively characteristic [6]. In the present case, white lesions appeared first, followed by erosions. No previous report has described changes in endoscopic findings over a long period in a single patient.

We observed that the area of whitish granular mucosa and erosions expanded and the appearance of lesions became more apparent over time while being exposed to lanthanum carbonate over 3 years. Interestingly, the lesion area spread on the atrophic area from near the pylorus toward the cardia. This spreading pattern seemed similar to the mucosal atrophy progress described by Takemoto et al. [7]. This finding suggests that there is a difference between normal vs. atrophic gastric mucosa in the susceptibility to the deposition of phosphorus and lanthanum. Ban et al. reported the deposition tended to occur in the gastric mucosa with the regenerative change, intestinal metaplasia, or foveolar hyperplasia [8] and this suggests the characteristics of background gastric mucosa can affect the lanthanum deposition. Given lanthanum carbonate is widely used for patients with dialysis and chronic kidney disease disrupts gastric and small intestinal epithelial tight junction (claudin-1) [9], there is a possibility that the increased epithelial permeability influence the deposition of lanthanum. Further studies investigating the factors causing differences in the susceptibility to the lanthanum deposition between different conditions of gastric mucosa could provide insights into the mechanisms of how lanthanum deposits in the tissue leading to a new development of preventive interventions. In addition, the deposition of lanthanum can happen at extra-gastric sites, such as duodenum [10]. Analyzing not only gastric but also extra-gastric lesions would be helpful to understand the underlying mechanism of the lanthanum deposition.

Meanwhile, the causality and the mechanism between lanthanum deposition and mucosal injury remains unestablished. Since the patient in this case report did not have an endoscopic examination before starting lanthanum carbonate, we could not assess the base-line gastric mucosa without the effect of the medication. Nonetheless, over-time endoscopic as well as histological observations clearly showed that the deposition of lanthanum and the number of histiocytes that infiltrated in the tissue were increasing and gastric inflammation became exacerbated during the treatment with lanthanum carbonate. Our findings imply the lanthanum deposition results in damaging the gastric mucosa. The studies to investigate the causal link and mechanisms of lanthanum deposition and mucosal injury are needed.

One study found that 85.7% of dialysis patients treated with lanthanum carbonate had deposition in the gastrointestinal mucosa [11]. Despite this high frequency, the long-term clinical effects of lanthanum deposition in the gastrointestinal mucosa are not yet clear. Whether chronic inflammation due to lanthanum deposition is a risk factor for the development of gastric cancer is also an important issue. There are reports that early gastric cancer was found in the gastric mucosa with lanthanum phosphate deposition [12, 13]. It is considered that the accumulation of cases and long-term observation are necessary.

## Data Availability

The datasets used and analyzed during the current study are available from the corresponding author on reasonable request.
